# Does Sensory Function Decline Independently or Concomitantly with Age? Data from the Baltimore Longitudinal Study of Aging

**DOI:** 10.1155/2016/1865038

**Published:** 2016-09-27

**Authors:** Shekhar K. Gadkaree, Daniel Q. Sun, Carol Li, Frank R. Lin, Luigi Ferrucci, Eleanor M. Simonsick, Yuri Agrawal

**Affiliations:** ^1^Department of Otolaryngology-Head & Neck Surgery, Johns Hopkins University School of Medicine, Baltimore, MD, USA; ^2^National Institute on Aging, National Institutes of Health, Baltimore, MD, USA

## Abstract

*Objectives*. To investigate whether sensory function declines independently or in parallel with age within a single individual.* Methods*. Cross-sectional analysis of Baltimore Longitudinal Study of Aging (BLSA) participants who underwent vision (visual acuity threshold), proprioception (ankle joint proprioceptive threshold), vestibular function (cervical vestibular-evoked myogenic potential), hearing (pure-tone average audiometric threshold), and Health ABC physical performance battery testing.* Results*. A total of 276 participants (mean age 70 years, range 26–93) underwent all four sensory tests. The function of all four systems declined with age. After age adjustment, there were no significant associations between sensory systems. Among 70–79-year-olds, dual or triple sensory impairment was associated with poorer physical performance.* Discussion*. Our findings suggest that beyond the common mechanism of aging, other distinct (nonshared) etiologic mechanisms may contribute to decline in each sensory system. Multiple sensory impairments influence physical performance among individuals in middle old-age (age 70–79).

## 1. Introduction

The peripheral sensory systems—including the visual, proprioceptive, auditory, and vestibular systems—provide feedback from the environment, facilitating interaction and engagement with the external world. Each sensory system may become impaired with age, contributing to increased isolation from the outside world. Age-related reductions in visual function, including in visual acuity, field of view, and contrast sensitivity, are universal in older individuals [1–3]. Additionally, proprioceptive function declines with age, with studies reporting differences in proprioceptive ability of around 50% in metrics such as joint deviation thresholds between older and younger adults [4–6]. Hearing is also well known to decline with age, with presbycusis a widespread phenomenon among individuals aged 80 and above [2, 7, 8]. A decline in vestibular physiologic function associated with age has also been well documented in a number of studies [9–12].

Although age-related decline in each of these peripheral sensory systems has been well established in the literature, it is unknown whether these systems tend to lose function concomitantly with age within a single individual. This is an important consideration as parallel decline in these systems would suggest a common pathophysiology, perhaps related to the biology of aging [13]. A few studies have examined the prevalence and impact of dual sensory impairments, typically involving hearing and vision and hearing and vestibular function [14–18]. One study that observed the common cooccurrence of vision and hearing loss found that these sensory impairments act multiplicatively and increase the risk of adverse health outcomes and mortality [2, 19]. Another study observed an association between hearing loss and vestibular loss and postulated that the inner ear cochlear and vestibular structures may have shared vulnerabilities to toxic exposures such as noise [20]. However, whether individuals with impairment in one peripheral sensory system have higher than expected risk of also developing impairment in other sensory systems is unknown.

In this study, we use data from the Baltimore Longitudinal Study of Aging (BLSA) to evaluate visual, proprioceptive, hearing, and vestibular function in relation to age and with respect to one another. We also explored how the presence of multiple sensory impairments influences physical performance across age categories. The BLSA is a large, prospective cohort of community-dwelling individuals spanning a wide age range that rigorously assesses each of these peripheral sensory systems. Findings from this study will provide insight into how changes in sensory function accrue with age, and the impact of multiple sensory impairments on physical disability.

## 2. Methods

### 2.1. Participants

Participants are enrolled in the BLSA, a prospective cohort study of the normative aging process conducted by the National Institute on Aging (NIA) Intramural Research Program. Healthy community-dwelling adults age ≥20 years are eligible to enroll in the BLSA [21]. At each visit, participants undergo a broad array of assessments and are followed longitudinally through the end of life. A total of 276 BLSA participants underwent tests of all four sensory systems: vision, proprioceptive, auditory, and vestibular between February 2013 and June 2014. The analytic sample was limited based on vestibular testing, which was only fully implemented on all participants in the BLSA in late 2013. Olfaction and taste are not measured in BLSA and therefore could not be evaluated in this study. Informed consent was obtained from all participants, and the study protocol has an ongoing approval with the institutional review board of the National Institute of Environmental Health Science, National Institutes of Health.

Trained interviewers administered detailed questionnaires on demographic and health-related data. Each participant underwent sensory evaluation as detailed below.

### 2.2. Visual Acuity Testing

A trained examiner conducted visual acuity testing using the Contrast Sensitivity Viewer-1000 Halogen Glare Test (CSV-1000 HGT) Early Treatment Diabetic Retinopathy Study (ETDRS) acuity chart (VectorVision, Greenville, OH). Participants were seated eight feet from the chart and a calibrated light at a constant level of 85 cd/m^2^ (candela per square meter) highlighted the row participants were prompted to read. Corrective lenses were permitted. Participants started reading a row where they could see all five letters clearly and progressed down the chart towards rows with smaller letters until they could correctly identify just two letters. This last line was considered to be their visual acuity level and was recorded as a Logarithm of the Minimal Angle of Resolution (LogMAR) score, where each line on the chart corresponds to a difference of 0.1 LogMAR. Standard visual acuity measurements of 20/10, 20/20, 20/40, and 20/200 correspond to LogMAR scores of −0.3, 0, 0.3, and 1.0 [21, 22]. For the sensory impairment analyses involving dichotomous measures, visual impairment was defined as any corrected LogMAR score greater than or equal to 0.3, corresponding to a 20/40 visual acuity [23].

### 2.3. Proprioceptive Testing

Trained examiners used customized equipment to quantitatively test proprioception [24]. The equipment consisted of two pedals: the right pedal controlled by a motor (BALDOR, Ft. Smith, AZ, USA) and the left pedal moved freely by the participant. Both pedals measured angle deviation from a baseline using potentiometers. For testing, participants were blindfolded and had their bare or stocking feet placed on the pedals, which were set at a neutral ankle angle of 100 degrees that would serve as the baseline for testing. The minimal angular displacement (degrees) of ankle deviation in the motorized pedal foot that could be detected by the participant was determined and recorded as the ankle proprioceptive threshold. The motorized pedal was moved at an angular speed of 0.3 degrees/second and followed a preset pattern of four trials. The lowest ankle threshold deviation angle (in degrees) from the four trials was considered the best threshold and was used as the measure of proprioception for this study [24]. For the sensory impairment analyses involving dichotomous measures, abnormal proprioception was defined as 2 standard deviations above the population average thresholds for men (1.00 degrees) and women (1.11 degrees) established previously in the BLSA [24]. This yielded cutoffs of >1.12 degrees for men and >1.25 degrees for women.

### 2.4. Audiometric Testing

Audiometric testing was performed by a trained examiner with the participant in a sound attenuating booth using an Interacoustics AD629 audiometer with ER3A insert earphones. Audiometric thresholds were obtained at frequencies 500 Hz to 8000 Hz. Pure-tone averages (PTA) were calculated as the mean thresholds in decibels Hearing Level (dB HL) at the frequencies of 0.5 KHz, 1 KHz, 2 KHz, 4 KHz, and 8 KHz in both ears [25]. The hearing threshold for each participant was calculated by averaging the mean thresholds across all frequencies in both ears, and those with an averaged hearing threshold of greater than 25 dB were considered to have impaired sensory function.

### 2.5. Vestibular Function Testing

Cervical vestibular-evoked myogenic potential (cVEMP) testing was used to assess vestibular function, specifically the function of the saccular end-organ of the vestibular system [26–28]. Participants were asked to lie at a 30-degree angle from the horizontal on the testing chair. Alcohol was used to cleanse the skin overlying the upper sternal area and both sternocleidomastoid (SCM) muscles, and electrodes were placed at these sites. A noninverting electrode was placed at the midpoint of the SCM muscle, an inverting electrode was placed at the sternoclavicular joint, and a ground electrode was placed at the upper sternum. Prior to applying the stimulus, participants were asked to lift their heads to provide a sample of background SCM activity. Audible stimuli were delivered through Audiocups noise canceling headphones from Amplivox (Eden Prairie, MN, USA). The stimulus was a 500 Hz, 125 dB Sound Pressure Level (SPL) tone burst, with a repetition rate of 5 Hz, a 1 ms rise/fall time, and a 2 ms plateau. In order for a cVEMP tracing to be valid, the background electromyography (EMG) signal was required to reach at least 30 mV over the 10 ms prior to the applied stimulus.

The cVEMP waveform consisted of a positive initial deflection followed by a negative deflection. The peak-to-peak amplitude was the voltage difference between the peak of the first positive deflection and the peak of the following negative deflection. The peak-to-peak cVEMP amplitude was divided by the background EMG signal to obtain the “corrected” peak-to-peak amplitude, which accounted for background level of muscle activity. Subjects with EMG recordings that lacked the initial characteristic positive deflection of the waveform were considered to have an absent cVEMP response and vestibular impairment for the purposes of this study.

The Health ABC Physical Performance Battery (PPB) score is a validated predictor of functional capacity, particularly useful for distinguishing higher levels of function [29, 30]. PPB testing consists of repeated chair stands, a six-meter normal walk, a six-meter narrow walk, and a standing series of balance testing (semi-tandem, full tandem, and single legged) [30]. Chair stand rate, average velocity for the normal walk, average velocity for the narrow walk, and the summed time held in each of the three balance stance tests were recorded and each result was divided by the maximal possible performance on the respective test, as determined by other studies [30–34]. Specifically, for chair stands, the maximal possible performance (MPP) value was 1 chair stand/second; for normal and narrow walks, the MPP was 2 m/s; for balance stance testing, the MPP was 90 seconds [30]. The ratios from each of these four domains, which ranged from 0 to 1, were summed for an ultimate score of 0 to 4, where 0 implied that none of the tests were performed successfully and 4 represented a perfect score [30]. In evaluating physical performance in this study, we used a cutoff score of 2.9, corresponding to the lowest third of physical performance tests, to indicate diminished physical performance.

### 2.6. Statistical Analysis

Simple linear regression models were used to evaluate the association between each sensory system and aging and between each of the sensory systems. For each bivariate association, a linear trend line and loess trend line were fitted to the graphical data. The loess trend line was created using loess regression, a locally weighted regression technique that provides a more detailed weighted trend line for grouped data segments [35]. Multiple linear regression analyses were used to evaluate the associations between sensory systems adjusted for age. Standardized regression coefficients were determined by calculating *z* scores in order to normalize each variable to be included in regression analysis. Estimating expected percentages of sensory impairments for multiple systems was calculated by multiplying the corresponding observed ratios of individual sensory impairments for a particular age category together. *p* values of less than 0.05 were considered statistically significant. All statistical analyses were performed using STATA 12.0 Statistical Software (College Station, TX, USA).

## 3. Results

**Figure 1:**
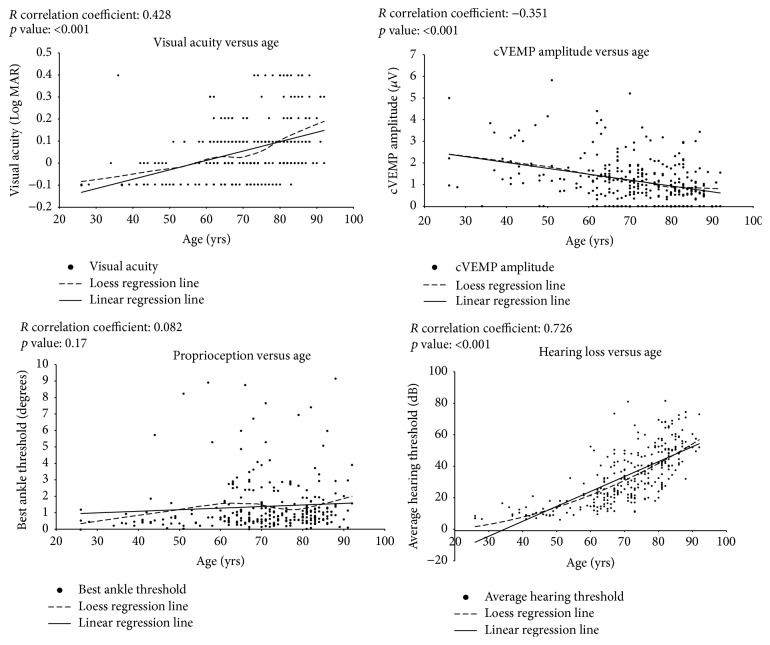
Individual associations between sensory system and age.

Between February 2013 and June 2014, 276 participants underwent visual, vestibular, hearing, and proprioceptive testing in the BLSA. Sociodemographic characteristics and mean sensory function of the study participants are presented in Table 1. The participants had a mean (SD) age of 70.3 (13.7) years, ranging from 26 to 93 years. Fifty-eight percent of study participants were female, and the distribution by race was 60.9% white, 28.6% black, 6.2% Asian or Pacific Islander, and 6.4% other race. Mean (SD) visual acuity for the study population was 0.06 (0.14) LogMAR. The mean (SD) ankle threshold to detect movement in the joint was 1.2 (1.1) degrees. The mean (SD) pure-tone average hearing threshold was 33.8 (17.8) dB. The mean (SD) cVEMP amplitude was 1.2 (1.1) *μ*V. We evaluated the associations between each of the peripheral sensory systems and age. As expected, we observed decline in sensory function with age, although these associations were only statistically significant for the visual, hearing, and vestibular systems (Figure 1). Visual acuity worsened with age (0.004 increase in LogMAR per year, *p* < 0.001). Hearing PTA thresholds increased with age (0.95 dB increase per year, *p* < 0.001), and cVEMP amplitude decreased with age (0.03 *μ*V per year, *p* < 0.001).

Next, we considered the mutual associations between each of the sensory systems in unadjusted and age-adjusted analyses (Table 2(a)). The unadjusted analyses indicate whether pairs of sensory systems decline concomitantly, and age-adjusted analyses suggest whether the observed associations in unadjusted analyses can be explained by a shared association with age. Visual acuity and vestibular function were associated with one another (*p* = 0.01), although neither system was associated with proprioceptive function in unadjusted analyses. Additionally, we observed a positive association between poorer hearing thresholds and both poorer visual acuity (*p* < 0.001) and poorer vestibular function (*p* < 0.001). In multivariate models that adjusted for age, none of the associations retained their statistical significance (Table 2(b)). This indicates that the functions of the sensory systems decline with age, but they do so independently of one another.

Further, we evaluated the proportion of individuals with multiple (2–4) concurrent sensory impairments across age categories in participants aged 60 or older (Table 3). We compared the observed prevalence of multiple sensory impairment with the expected prevalence based on compounded probabilities of multiple impairments under the assumption of independence (i.e., sensory impairments occur independently). Overall, we observed an increase in the prevalence of multiple sensory impairments with increasing age category. Among the participants aged 80 years or older, 43.6% had dual sensory impairment and the most prevalent dual sensory impairment was hearing and vestibular loss (17.2%). Eighteen percent of participants aged 80 years and older had triple sensory impairment and the most prevalent triple sensory impairment was vision, proprioception, and hearing loss (8.0%). Only two participants had quadruple sensory impairments and both were over age 80. Notably, observed probabilities for dual sensory impairments were often lower than expected under an assumption of independence.

Finally, we explored the physical performance associated with single versus multiple sensory impairments across age categories in participants aged 60 or older (Table 3). Overall, we observed that participants aged 60–69 had normal Health ABC physical performance scores, while participants aged ≥80 had the lowest tertile Health ABC performance scores. Sensory impairments influenced physical performance in the middle old-age category (age 70–79). Individuals with single vision or proprioceptive impairments had poor physical performance, whereas individuals with single hearing or vestibular impairments had normal physical performance. Dual vision and proprioceptive, vestibular and hearing, and vision and hearing impairments were associated with poor physical performance, and all triple sensory impairments were associated with poor physical performance.

## 4. Discussion

Our results demonstrate that while visual acuity, proprioceptive function, hearing, and vestibular function all decline with age, these sensory systems also decline through other mechanisms unique to each sensory system. After adjustment for age in regression models, there were no significant associations between any of the sensory systems. This observation suggests that, beyond the common mechanism of aging, other distinct (nonshared) etiologic mechanisms may contribute to the decline in each of these sensory systems. For instance, low antioxidant levels and vitamin deficiencies may disproportionately contribute to vision loss, while noise exposure and ototoxic medications may uniquely affect hearing [36–39]. The vestibular system may be specifically targeted by certain viral infections and aminoglycoside antibiotics, while proprioception may be particularly affected by diabetes [4, 40, 41]. Our findings suggest that the probability that sensory impairments cooccur within an individual is not greater than that expected by chance; however, an alternate explanation for these findings is that age itself may explain all variability in sensory impairment.

This study is among the first to evaluate the multiple sensory decline that occurs with aging. Growing evidence suggests that progressive loss of sensory input leads to increased isolation from the external world and presages important geriatric outcomes such as declining physical and social activity, mobility disability, falls, and dementia [42, 43]. Although sensory impairments appear to occur through independent age-related mechanisms, multiple impairments are more likely to accrue in older individuals because the prevalence of each impairment increases with age.

We also observed that single versus multiple sensory impairments impact physical performance among participants in “middle” old-age (age 70–79). Younger participants had normal physical performance and older participants had impaired physical performance regardless of sensory function. This suggests that other key factors, such as muscle strength and reaction time, contribute to physical performance and decline with age. Our findings suggest the particular importance of vision and proprioception in physical performance, given that single impairments in these sensory systems were associated with impaired physical performance. Most dual sensory impairments and all triple sensory impairments among 70–79-year-olds were associated with poor physical performance. Further studies in larger samples will be needed to establish which thresholds and combinations of sensory impairments result in poorer physical and functional status.

Very few studies have investigated multiple sensory impairments. One nationally representative study of the US population found that 11.3% of adults aged 80 or older had concurrent vision and hearing loss, similar to the 9.2% prevalence of dual vision and hearing impairment observed in this sample [2]. A longitudinal study in women found that individuals with dual vision and hearing loss were significantly more likely to experience incident cognitive decline [14]. Another population-based longitudinal study in Australia found that dual vision and hearing impairment at baseline was associated with a significantly increased risk of 10-year all-cause mortality [19]. Of note, in both studies, individuals with single impairments did not have increased risk of either cognitive decline or death relative to individuals with no sensory impairments. These data underscore the hazards of multiple sensory impairments with respect to adverse geriatric outcomes. Future studies will be needed to also consider how multiple impairments including vestibular and proprioceptive function affect morbidity and mortality risk. Interestingly, we observed that the prevalence of multiple sensory impairments (specifically dual) was lower than expected based on calculated probabilities. Whether enhancement in a modality of sensory function may occur in the context of loss of another sensory modality remains to be explored.

This study has several limitations. For the sake of simplicity, only a single measure was used to represent each of the sensory systems. However, each of the sensory systems has multiple dimensions; for example, vision encompasses not only visual acuity but also visual fields and contrast sensitivity, and vestibular function includes not only saccular function but also the function of the semicircular canals. Further studies involving larger cohorts will be needed to evaluate the parallel versus interrelated trajectories of each of these subdomains of sensory function. Moreover, we did not consider olfaction or taste sensation, given that these systems are not evaluated in the BLSA. Finally, the BLSA cohort is a highly selected sample that is healthier than the general US population. As such, findings from this analysis may not be applicable to other populations with higher rates of disease burden. For example, while this study suggests that there is no concomitant sensory decline of multiple sensory systems beyond the effects of aging in healthy individuals, cohorts with significant neuropathology, such as Alzheimer's Disease, are known to have linked impairment in olfaction loss, hearing loss, and vision loss [25, 44, 45]. Further data collection will be required to evaluate how the chemical sensory systems are impaired with age relative to the other sensory systems in participants with significant disease pathology.

## 5. Conclusion

In conclusion, we observed that while peripheral sensory function declines with age, once the effect of age is accounted for sensory decline occurs through independent mechanisms. Nevertheless the probability of multiple sensory impairments increases with age, given that each impairment is more likely to occur at older ages. Further investigation is needed to evaluate whether different combinations of sensory impairments confer specific risks and how these risks can be mitigated.

## Figures and Tables

**Table 1:** Demographic characteristics and baseline sensory function, Baltimore Longitudinal Study of Aging 2013-4.

Characteristics	No (%)/mean (±SD^a^)
Total participants	276
Age (years)	70.3 (±13.7)
Sex	
Male	116 (42.0%)
Female	160 (58.0%)
Race	
White, non-Hispanic	168 (60.9%)
Black, non-Hispanic	79 (28.6%)
Asian or Pacific Islander	17 (6.2%)
Other	12 (6.4%)
Best proprioception threshold (degrees)	1.4 (±1.6)
Visual acuity (LogMAR^b^)	0.06 (±0.14)
Hearing threshold (dB)	33.8 (±17.8)
cVEMP^c^ amplitude (*μ*V)	1.2 (±1.1)

^a^SD: standard deviation.

^b^LogMAR: Logarithm of the minimal angle of resolution.

^c^cVEMP: cervical vestibular-evoked myogenic potential.

**(a):** 

	Vestibular function	Proprioceptive threshold	Visual acuity	Hearing threshold
Vestibular function		−0.02 (−0.14, 0.10) *p* = 0.73^†^	−0.13 (−0.25, −0.02) *p* = 0.026^†^	−0.24 (−0.36, −0.13) *p* < 0.001^†^
Proprioceptive threshold	−0.01 (−0.09, 0.07) *p* = 0.78		−0.02 (−0.14, 0.09) *p* = 0.68^†^	0.06 (−0.06, 0.18) *p* = 0.32^†^
Visual acuity	−1.21 (−2.1, −0.28) *p* = 0.01^‡^	−0.29 (−1.7, 1.1) *p* = 0.68		0.33 (0.22, 0.44) *p* < 0.001^†^
Hearing threshold	−0.014 (−0.02, −0.01) *p* < 0.001^‡^	0.005 (−0.01, 0.02) *p* = 0.32	0.002 (0.001, 0.003) *p* < 0.001^‡^	

Regression coefficients refer to change in the column variable associated with 1 unit change in row variable for bottom half of table. † represents normalized regression coefficients corresponding to bottom half of table. 95% confidence intervals are included in parentheses. The units for the variables are vestibular function (VOR gain ratio), proprioceptive threshold (degrees), visual acuity (LogMAR), and hearing threshold (dB).

‡ = *p* value < 0.05.

**(b):** 

	Vestibular function	Proprioceptive threshold	Visual acuity	Hearing threshold
Vestibular function		0.008 (−0.10, 0.12) *p* = 0.89^†^	0.018 (−0.11, 0.14) *p* = 0.77^†^	0.019 (−0.14, 0.18) *p* = 0.82^†^
Proprioceptive threshold	0.009 (−0.07, 0.08) *p* = 0.82		−0.074 (−0.20, 0.06) *p* = 0.27^†^	0.001 (−0.17, 0.17) 0.99^†^
Visual acuity	−0.005 (−0.99, 0.98) *p* = 0.99	−0.86 (−2.4, 0.67) *p* = 0.27		0.036 (−0.12, 0.19) *p* = 0.65^†^
Hearing threshold	0.001 (−0.01, 0.01) *p* = 0.83	0.001 (−0.02, 0.02) *p* = 0.99	0.001 (−0.001, 0.001) *p* = 0.65	

Regression coefficients refer to the change in the column variable associated with 1 unit change in row variable for bottom half of table. † represents normalized regression coefficients corresponding to bottom half of table. 95% confidence intervals are included in parentheses. The units for the variables are vestibular function (VOR gain ratio), proprioceptive threshold (degrees), visual acuity (LogMAR), and hearing threshold (dB).

**Table 3:** Number of impaired sensory systems in study participants by age and Health ABC score.

Number of impairments	Sensory system	Age categories					
		60–69 (*N* = 68)		70–79 (*N* = 78)		80+ (*N* = 87)	
		Observed *N* (%)^‡^	Expected *N* (%)	Observed *N* (%)^‡^	Expected *N* (%)	Observed *N* (%)^‡^	Expected *N* (%)
Any	Hearing (Hear)	25/68 (36.8)^†^		60/78 (76.9)^†^		84/87 (97.0)^#^	
	Vestibular (Vest)	11/68 (16.2)^†^		14/78 (17.9)^†^		27/87 (31.0)^#^	
	Vision (Vis)	3/68 (4.4)^†^		5/78 (6.4)^#^		20/87 (23.0)^#^	
	Proprioceptive (Pro)	24/68 (35.3)^†^		25/78 (32.1)^#^		30/87 (34.5)^#^	

Two	Vest + Vis	0/68 (0)^‡^	<1/68 (0.71)	1/78 (1.3)^†^	<1/78 (1.1)	0/87 (0)^‡^	6.2/87^*∗*^ (7.1)
	Vest + Prop	3/68 (4.4)^†^	3.9/68 (5.7)	0/78 (0)^‡^	4.5/78^*∗*^ (5.7)	1/87 (1.1)^#^	9.3/87 (10.7)
	Vis + Prop	1/68 (1.5)^†^	1.1/68 (1.6)	1/78 (1.3)^#^	1.6/78 (2.1)	0/87 (0)^‡^	6.9/87^*∗∗*^ (7.9)
	Vest + Hear	3/68 (4.4)^†^	4.1/68 (6.0)	6/78 (7.7)^#^	11/78 (13.8)	15/87 (17.2)^#^	26/87^*∗*^ (30.1)
	Vis + Hear	0/68 (0)^‡^	1.1/68 (1.6)	1/78 (1.3)^#^	3.8/78 (4.9)	8/87 (9.2)^†^	19/87^*∗*^ (22.3)
	Prop + Hear	5/68 (7.4)^#^	8.8/68 (13.0)	17/78 (21.8)^†^	19/78 (24.7)	14/87 (16.1)^#^	29/87^*∗*^ (33.5)

Three	Vest+ Vis + Prop	0/68 (0)^‡^	<1/68 (0.25)	0/78 (0)^‡^	<1/78 (0.36)	0/87 (0)^‡^	2.1/87 (2.5)
	Vest + Vis + Hear	0/68 (0)^‡^	<1/68 (0.26)	0/78 (0)^‡^	<1/78 (0.88)	3/87 (3.4)^#^	6/87 (6.9)
	Vis + Prop + Hear	0/68 (0)^‡^	<1/68 (0.57)	2/78 (2.6)^#^	1.2/78 (1.6)	7/87 (8.0)^#^	6.7/87 (7.7)
	Prop + Hear + Vest	2/68 (2.9)^†^	1.4/68 (2.1)	3/78 (3.8)^#^	3.4/78 (4.4)	6/87 (6.9)^#^	9.0/87 (10.4)

Four	Vest + Vis + Prop + Hear	0/68 (0)^‡^	<1/68 (0.09)	0/78 (0)^‡^	<1/78 (0.28)	2/87 (2.3)^#^	2.1/87 (2.4)

Greater than or equal to Health ABC score of 2.9 marked as †; below cutoff marked as #.

Single or double asterisk signifies observed and expected probabilities being significantly different using Chi-squared analysis (^*∗*^ *p* < 0.05) (^*∗∗*^ *p* < 0.01).
